# A multicenter mixed-effects model for inference and prediction of 72-h return visits to the emergency department for adult patients with trauma-related diagnoses

**DOI:** 10.1186/s13018-020-01863-8

**Published:** 2020-08-14

**Authors:** Ehsan Yaghmaei, Louis Ehwerhemuepha, William Feaster, David Gibbs, Cyril Rakovski

**Affiliations:** 1 CHOC Children’s, Orange, CA 92868 USA; 2 Schmid College of Science & Technology, Chapman University, Orange, CA USA

**Keywords:** Emergency department, Return visits, Trauma, Adult medicine

## Abstract

**Objective:**

Emergency department (ED) return visits within 72 h may be a sign of poor quality of care and entail unnecessary use of healthcare resources. In this study, we compare the performance of two leading statistical and machine learning classification algorithms, and we use the best performing approach to identify novel risk factors of ED return visits.

**Methods:**

We analyzed 3.2 million ED encounters with at least one diagnosis under “injury, poisoning and certain other consequences of external causes” and “external causes of morbidity.” These encounters included patients 18 years or older from across 128 emergency room facilities in the USA. For each encounter, we calculated the 72-h ED return status and retrieved 57 features from demographics, diagnoses, procedures, and medications administered during the process of administration of medical care. We implemented a mixed-effects model to assess the effects of the covariates while accounting for the hierarchical structure of the data. Additionally, we investigated the predictive accuracy of the extreme gradient boosting tree ensemble approach and compared the performance of the two methods.

**Results:**

The mixed-effects model indicates that certain blunt force and non-blunt trauma inflates the risk of a return visit. Notably, patients with trauma to the head and patients with burns and corrosions have elevated risks. This is in addition to 11 other classes of both blunt force and non-blunt force traumas. In addition, prior healthcare resource utilization, patients who have had one or more prior return visits within the last 6 months, prior ED visits, and the number of hospitalizations within the 6 months are associated with increased risk of returning to the ED after discharge. On the one hand, the area under the receiver characteristic curve (AUROC) of the mixed-effects model was 0.710 (0.707, 0.712). On the other hand, the gradient boosting tree ensemble had a lower AUROC of 0.698 CI (0.696, 0.700) on the independent test model.

**Conclusions:**

The proposed mixed-effects model achieved the highest known AUC and resulted in the identification of novel risk factors. The model outperformed one of the leading machine learning ensemble classifiers, the extreme gradient boosting tree in terms of model performance. The risk factors we identified can assist emergency departments to decrease the number of unplanned return visits within 72 h.

## Introduction

Emergency departments across the USA are continually working on improving the quality of care as measured by health outcomes of patients, overall patient experience, and reduction in cost to both patients and facilities. These emergency department (ED) facilities are seeing annual increases in patient census that may impact the quality of care [1–3]. This increase in ED utilization is coupled with existing issues of overcrowding to exacerbate the challenges of providing a high quality of care and reducing both morbidity and mortality [4–11]. These challenges are complex and multifaceted and are further worsened by return visits to the ED that are avoidable. Consequently, the rate of return visits to the ED within 72 h of a previous discharge is being used as a metric for quality of care in the ED [12–15]. Return visits to the ED may be reflective of poor quality of care but may also be caused by latent illnesses and misdiagnoses [16], unrelated new problems [17], perceived inability to access timely follow-up care, and patient uncertainty or fear about disease progression [18].

Patients with trauma/injuries have particularly high rates of potentially unnecessary return visits, with over 43.1% of corresponding revisits estimated as being avoidable in this group [12]. It is therefore important to understand and address the factors associated with return visits within this population. Several attempts have been made to address this issue, including studies focused on the role of patient demographics and socioeconomic status, mode of transportation, and level of trauma activation [19]. Further attempts have been made with a focus on patients with head injuries [20].

In this study, we specifically explored new variables in search of novel risk factors associated with ED returns for patients with trauma-related codes as captured by the International Classification of Diseases, Tenth Revision (ICD-10-CM) codes of S00-T79 (injury, poisoning and certain other consequences of external causes) and V00-Y99 (external causes of morbidity). There have been no comprehensive studies on the prediction of ED return visits among patients with trauma/injuries. Existing studies analyzed risk factors for presentation to the ED after the discharge of trauma patients from the hospital [21] and the effect of head trauma on risk of ED return within 72 h [20]. The objective of this study is to address this important problem and explore novel risk factors of ED revisits and design a corresponding prediction model. We compared the performance of advanced statistical methods and a high accuracy machine learning algorithm to determine the optimal classification model. We provide an assessment of model performance with recommendations on the potential implementation of the corresponding predictive models in the ED. The new variables we considered include several measures of current and past healthcare resource utilization that have been found to be associated with the related problem of hospital readmission [22–24].

## Methods

This study was approved by CHOC Children’s Hospital Institutional Review Board (IRB 180857).

### Study design and setting

The data source for the study is the Cerner Health Facts database (referred to as Health Facts DB from here on). The Health Facts DB consists of data captured by the Cerner Corporation from over 100 US healthcare systems and over 650 facilities (in 2018) that is aggregated and organized into consumable datasets to facilitate research and reporting. It consists of clinical database tables that include information on ED visits, diagnoses, and medications. The descriptive and predictive multi-center models developed in this study were built using a subset of data from the database based on a priori inclusion criteria. An extensive analysis of a prior version of the database has been conducted with recommendations on its use [25].

### Selection of participants, measurements, and outcomes

We retrieved emergency department admission on patients 18 years or older from ED facilities in the USA from the Health Facts DB. We included EDs that contributed to the key database tables for the study (encounters, diagnoses, and medications tables) and have seen a large number of patients (set a priori at 10,000). These inclusion criteria ensured both the exclusion of potentially noisy data and the inclusion of large sample centers. We included multiple index encounters and revisits within 72 h for individual patients, and each encounter that was itself a revisit within 72 h was treated as an index encounter for estimating subsequent revisit to the ED. We included demographic variables as well as proxies for socioeconomic status, prior ED and hospital utilization variables, diagnoses, and the total number of medications administered during the ED visit.

### Analysis

**Fig. 1:**
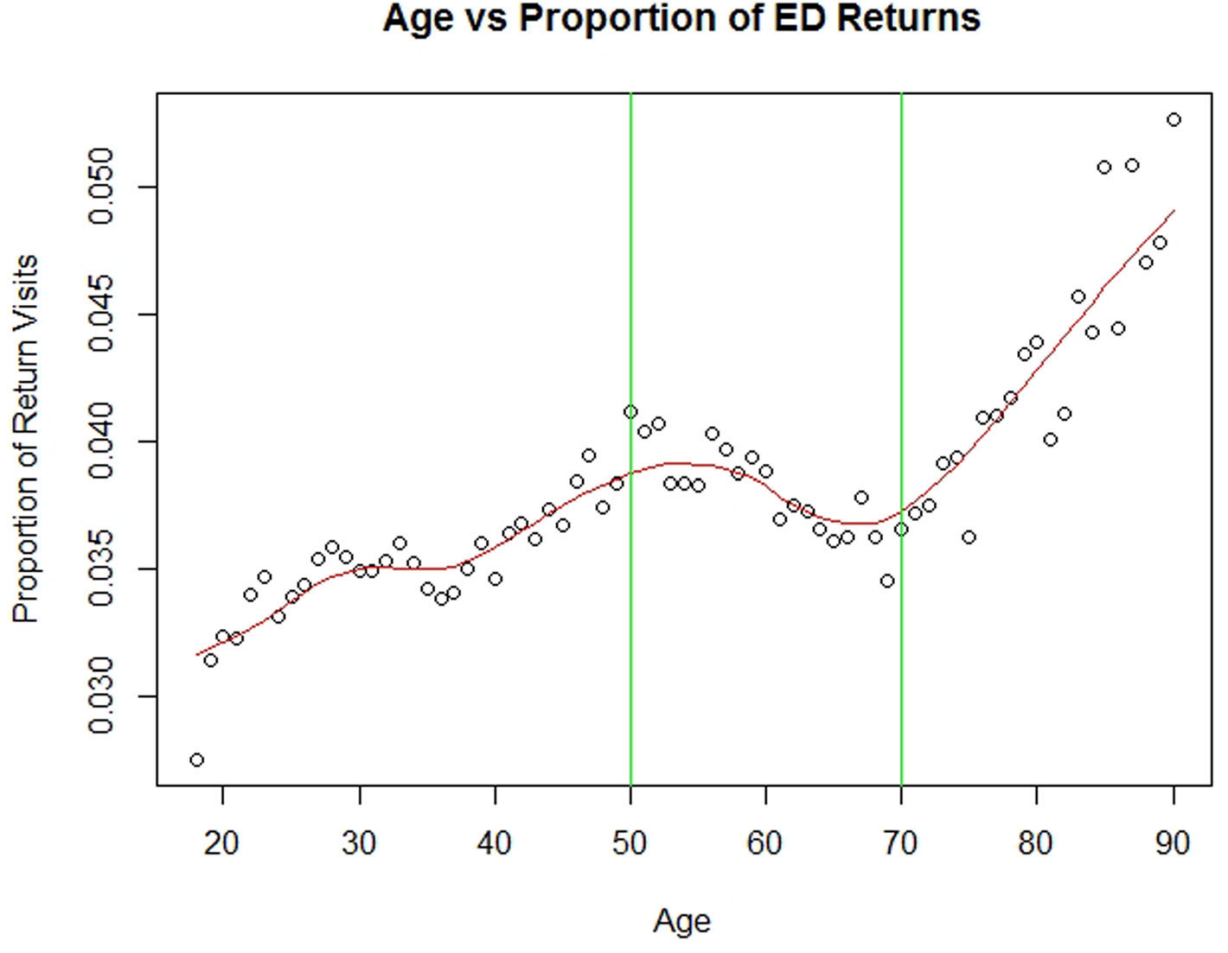
Categorization of age. Age vs. proportion of ED returns

We categorized the ages of the patients based on the distribution of readmission rates by age, as shown in Fig. 1. We excluded very sparse variables (defined a priori as having less than 1000 responses) to prevent issues with statistical separation [26]. Sparse outcomes or analysis of rare events requires exact statistical tests [27].

We assessed multicollinearity by estimating the generalized variance inflation factor [28, 29] (GVIF) of the variables. In a stepwise process, we excluded the variable with the highest GVIF and reassessed multicollinearity until the GVIF of all variables kept is below 4—a rule of thumb threshold based on the previous studies [23]. We randomly split the data into two: 50% for model and the other 50% for evaluating model performance. We implemented a mixed-effects logistic regression model and gradient boosting tree ensemble [30, 31]. We conducted variable selection on the random intercept model using stepwise minimization of the Akaike Information Criteria and grid search for hyperparameter tuning on the gradient boosting algorithm. We assessed model performance using the area under the receiver operator characteristic curve (AUROC) and sensitivity and positive predictive value at a specificity of 0.90. Analyses were carried out using Apache Spark [32, 33], the R Statistical Computing Programming Language [34], and Python [35].

## Results

### Characteristics of study subjects

A total of 128 ED facilities met the inclusion criteria resulting in 2.2 million patients and 3.2 encounters. Each facility contributed data from different periods of time between 2000 and 2017, and the average number of years of data from the facilities is 7.6 years with a standard deviation of 2.7 years. There were 64.8, 23.8, and 11.4% of patients with ages 18–49, 50–69, and 70 years or older, respectively. Note that the process of deidentification included a requirement to specify the age of patients older than 90 years as 90 to reduce the possibility of reidentification of patients by age. Patient sex consisted of 50.9% males, 49.0% females, and the remaining of unknown sex, while 68.3, 17.5, and 14.2% were Caucasian, African American or Black, and other races and ethnicities. The overall rate of 72-h return visit to the ED is 0.037.

There were 1.6 million encounters in the training dataset after splitting the data into two halves. In Tables 1 and 2, we provide the summary statistics on the training dataset, which includes all 57 variables we considered during model development. We excluded variables capturing surgical procedures on the endocrine, hemic/lymphatic, and mediastinum/diaphragm systems due to sparsity and potential problems with statistical separation and multicollinearity.

**Table 1:** Summary statistics: demographics and trauma/injuries

Variable	Levels	No return visit (72 h)	Had a return visit (72 h)	*p* value (chi-squared or *t* test)
		*n* (%) or mean (sd)	*n* (%) or mean (sd)	
Age, years	18–49	985,654 (64.96)	35,628 (61.55)	< 0.001
	50–69	359,481 (23.69)	14,395 (24.87)	
	70 or older	172,214 (11.35)	7861 (13.58)	
Race and/or ethnicity	Caucasian	1,037,221 (68.36)	38,904 (67.21)	< 0.001
	Hispanic	31,535 (2.08)	964 (1.67)	
	Black	266,147 (17.54)	10,178 (17.58)	
	Asian	19,092 (1.26)	508 (0.88)	
	Native American	43,271 (2.85)	3362 (5.81)	
	Other	120,083 (7.91)	3968 (6.86)	
Sex	Female	774,225 (51.02)	27,336 (47.23)	< 0.001
	Male	741,267 (48.85)	30,518 (52.72)	
	Other/undisclosed	1857 (0.12)	30 (0.05)	
Length of stay (hours)	0 to 5	1,408,772 (92.84)	51,538 (89.04)	< 0.001
	6 to 11	91,484 (6.03)	4905 (8.47)	
	12 or more	17,093 (1.13)	1441 (2.49)	
Payer	Commercial other	487,429 (32.12)	16,044 (27.72)	< 0.001
	Medicare or Medicaid	491,449 (32.39)	23,161 (40.01)	
	Other governmental	57,319 (3.78)	2224 (3.84)	
	Self-pay	267,644 (17.64)	10,080 (17.41)	
	Others	213,508 (14.07)	6375 (11.01)	
History of hospital visits	0	1,421,064 (93.65)	49,523 (85.56)	< 0.001
	1	70,773 (4.66)	5155 (8.91)	
	2	16,400 (1.08)	1712 (2.96)	
	3 or more	9112 (0.60)	1494 (2.58)	
Is index ED visit a revisit (within 72 h) of a previous encounter?	No	1,463,245 (96.43)	50,429 (87.12)	< 0.001
	Yes	54,104 (3.57)	7455 (12.88)	
Previous ED visit	0	1,051,707 (69.31)	27,767 (47.97)	< 0.001
	1	252,012 (16.61)	10,311 (17.81)	
	2	95,731 (6.31)	5460 (9.43)	
	3 or more	117,899 (7.77)	14,346 (24.78)	
History of return visits	0	1,459,191 (96.17)	47,481 (82.03)	< 0.001
	1	39,372 (2.59)	4317 (7.46)	
	2	9389 (0.62)	1829 (3.16)	
	3 or more	9397 (0.62)	4257 (7.35)	
Number of medications	Less than 4	1,423,293 (93.80)	52,342 (90.43)	< 0.001
	4 or more	94,056 (6.20)	5542 (9.57)	
Season	Winter	324,662 (21.40)	12,281 (21.22)	0.003
	Spring	364,282 (24.01)	13,878 (23.98)	
	Summer	437,620 (28.84)	17,082 (29.51)	
	Fall	390,785 (25.75)	14,643 (25.30)	
Injuries and trauma				
Injuries to the head (S00-S09)	No	1,178,863 (77.69)	42,170 (72.85)	< 0.001
	Yes	338,486 (22.31)	15,714 (27.15)	
Injuries to the neck (S10-S19)	No	1,323,354 (87.21)	50,420 (87.11)	0.442
	Yes	193,995 (12.79)	7464 (12.89)	
Injuries to the thorax (S20-S29)	No	1,354,255 (89.25)	52,409 (90.54)	< 0.001
	Yes	163,094 (10.75)	5475 (9.46)	
Injuries to the abdomen, lower back, lumbar spine, pelvis, and external genitals (S30-S39)	No	1,336,251 (88.06)	51,054 (88.20)	0.326
	Yes	181,098 (11.94)	6830 (11.80)	
Injuries to the shoulder and upper arm (S40-S49)	No	1,380,266 (90.97)	53,145 (91.81)	< 0.001
	Yes	137,083 (9.03)	4739 (8.19)	
Injuries to the elbow and forearm (S50-S59)	No	1,418,621 (93.49)	53,603 (92.60)	< 0.001
	Yes	98,728 (6.51)	4281 (7.40)	
Injuries to the wrist, hand, and fingers (S60-S69)	No	1,242,857 (81.91)	47,690 (82.39)	0.003
	Yes	274,492 (18.09)	10,194 (17.61)	
Injuries to the hip and thigh (S70-S79)	No	1,438,356 (94.79)	54,311 (93.83)	< 0.001
	Yes	78,993 (5.21)	3573 (6.17)	
Injuries to the knee and lower leg (S80-S89)	No	1,297,862 (85.53)	50,337 (86.96)	< 0.001
	Yes	219,487 (14.47)	7547 (13.04)	
Injuries involving multiple body regions(T07)	No	1,467,511 (96.72)	55,833 (96.46)	< 0.001
	Yes	49,838 (3.28)	2051 (3.54)	
Injury of unspecified body region (T14-T14)	No	1,438,869 (94.83)	54,732 (94.55)	0.004
	Yes	78,480 (5.17)	3152 (5.45)	
Effects of foreign body entering through natural orifice (T15-T19)	No	1,491,654 (98.31)	57,218 (98.85)	< 0.001
	Yes	25,695 (1.69)	666 (1.15)	
Burns and corrosions (T20-T32)	No	1,495,027 (98.53)	56,775 (98.08)	< 0.001
	Yes	22,322 (1.47)	1109 (1.92)	
Poisoning by, adverse effect of, and underdosing of drugs, medicaments, and biological substances (T36-T50)	No	1,478,655 (97.45)	55,611 (96.07)	< 0.001
	Yes	38,694 (2.55)	2273 (3.93)	
Toxic effects of substances chiefly nonmedicinal as to source (T51-T65)	No	1,494,884 (98.52)	56,899 (98.30)	< 0.001
	Yes	22,465 (1.48)	985 (1.70)	
Other and unspecified effects of external causes (T66-T78)	No	1,459,709 (96.20)	54,995 (95.01)	< 0.001
	Yes	57,640 (3.80)	2889 (4.99)	
Complications of surgical and medical care, not elsewhere classified (T80-T88)	No	1,488,928 (98.13)	55,404 (95.72)	< 0.001
	Yes	28,421 (1.87)	2480 (4.28)	
Injuries to the ankle and foot (S90-S99)	No	1,289,905 (85.01)	50,655 (87.51)	< 0.001
	Yes	227,444 (14.99)	7229 (12.49)

**Table 2:** Summary statistics: other diagnoses/comorbidities and surgical procedures

Variable	Levels	No return visit (72 h)	Had a return visit (72 h)	*p* value (chi-squared or *t* test)
		*n* (%) or mean (sd)	*n* (%) or mean (sd)	
Other diagnoses/comorbidities				
Certain infectious and parasitic diseases (A00-B99)	No	1,501,709 (98.97)	57,020 (98.51)	< 0.001
	Yes	15,640 (1.03)	864 (1.49)	
Neoplasms (C00-D49) (excluding encounters for chemotherapy)	No	1,510,526 (99.55)	57,523 (99.38)	< 0.001
	Yes	6823 (0.45)	361 (0.62)	
Diseases of the blood and blood-forming organs and certain disorders involving the immune mechanism (D50-D89)	No	1,504,241 (99.14)	57,041 (98.54)	< 0.001
	Yes	13,108 (0.86)	843 (1.46)	
Endocrine, nutritional, and metabolic diseases (E00-E89)	No	1,376,422 (90.71)	51,121 (88.32)	< 0.001
	Yes	140,927 (9.29)	6763 (11.68)	
Mental, behavioral, and neurodevelopmental disorders (F01-F99)	No	1,296,066 (85.42)	45,219 (78.12)	< 0.001
	Yes	221,283 (14.58)	12,665 (21.88)	
Disease of the nervous system (G00-G99)	No	1,417,394 (93.41)	52,214 (90.20)	< 0.001
	Yes	99,955 (6.59)	5670 (9.80)	
Diseases of the eye and adnexa (H00-H59)	No	1,474,791 (97.20)	56,370 (97.38)	0.007
	Yes	42,558 (2.80)	1514 (2.62)	
Diseases of the circulatory system (I00-I99)	No	1,330,196 (87.67)	48,567 (83.90)	< 0.001
	Yes	187,153 (12.33)	9317 (16.10)	
Diseases of the respiratory system (J00-J99)	No	1,449,050 (95.50)	54,362 (93.92)	< 0.001
	Yes	68,299 (4.50)	3522 (6.08)	
Diseases of the digestive system (K00-K95)	No	1,453,950 (95.82)	54,637 (94.39)	< 0.001
	Yes	63,399 (4.18)	3247 (5.61)	
Diseases of the skin and subcutaneous tissue (L00-L99)	No	1,456,934 (96.02)	53,432 (92.31)	< 0.001
	Yes	60,415 (3.98)	4452 (7.69)	
Diseases of the musculoskeletal system and connective tissue (M00-M99)	No	963,184 (63.48)	39,179 (67.69)	< 0.001
	Yes	554,165 (36.52)	18,705 (32.31)	
Disease of the genitourinary system (N00-N99)	No	1,433,691 (94.49)	53,793 (92.93)	< 0.001
	Yes	83,658 (5.51)	4091 (7.07)	
Pregnancy, childbirth, and the puerperium (O00-O9A)	No	1,508,641 (99.43)	57,549 (99.42)	0.902
	Yes	8708 (0.57)	335 (0.58)	
Congenital malformations, deformations, and chromosomal abnormalities (Q00-Q99)	No	1,514,720 (99.83)	57,788 (99.83)	0.711
	Yes	2629 (0.17)	96 (0.17)	
Symptoms, signs, and abnormal clinical and laboratory findings, not elsewhere classified (R00-R99)	No	1,210,410 (79.77)	42,401 (73.25)	< 0.001
	Yes	306,939 (20.23)	15,483 (26.75)	
External causes of morbidity (V00-Y99)	No	389,533 (25.67)	15,725 (27.17)	< 0.001
	Yes	1,127,816 (74.33)	42,159 (72.83)	
Factors influencing health status and contact with health services (Z00-Z99)	No	1,198,725 (79.00)	42,590 (73.58)	< 0.001
	Yes	318,624 (21.00)	15,294 (26.42)	
Surgical procedures				
Integumentary surgery (CPT4: 10030-19499)	No	1,425,628 (93.96)	53,469 (92.37)	< 0.001
	Yes	91,721 (6.04)	4415 (7.63)	
Musculoskeletal surgery (CPT4: 20100-29999)	No	1,460,822 (96.27)	56,089 (96.90)	< 0.001
	Yes	56,527 (3.73)	1795 (3.10)	
Respiratory surgery (CPT4: 30000-32999)	No	1,516,932 (99.97)	57,790 (99.84)	< 0.001
	Yes	417 (0.03)	94 (0.16)	
Cardiovascular surgery (CPT4: 33010-37799)	No	1,495,828 (98.58)	56,739 (98.02)	< 0.001
	Yes	21,521 (1.42)	1145 (1.98)	
Digestive surgery (CPT4: 40490-49999)	No	1,512,836 (99.70)	57,767 (99.80)	< 0.001
	Yes	4513 (0.30)	117 (0.20)	
Urinary/reproductive system surgery (CPT4: 50010-58999)	No	1,515,308 (99.87)	57,740 (99.75)	< 0.001
	Yes	2041 (0.13)	144 (0.25)	
Nervous system surgery (CPT4: 61000-64999)	No	1,515,677 (99.89)	57,798 (99.85)	0.008
	Yes	1672 (0.11)	86 (0.15)	
Eye/ocular surgery (CPT4: 65091-68899)	No	1,515,061 (99.85)	57,833 (99.91)	< 0.001
	Yes	2288 (0.15)	51 (0.09)	
Auditory surgery (CPT4: 69000-69979)	No	1,516,050 (99.91)	57,867 (99.97)	< 0.001
	Yes	1299 (0.09)	17 (0.03)	

### Main results

Our results indicate that the highest risk factors attributable to the type of trauma/injuries include certain early complications of trauma such as embolisms and traumatic compartment syndrome (ICD 10 CM: T79); burns and corrosions (T20-T32); certain effects of external causes such as hypothermia, asphyxiation, and abuse (T66-T78); poisoning due to medical and biological substances (T36-T50); and injuries to the head (S00-S09). Patients with early complications of trauma have 120% increase in odds of return visit; patients with burns and corrosions, effects of external causes (such as hypothermia, asphyxiation, and abuse), poisoning, and injuries to the head have an increase in odds of 55, 54, 35, and 29% respectively. We found eight additional categories associated with increased odds of a return visit. Patients suffering from the toxic effects of nonmedicinal sources such as alcohol, carbon monoxide, and venom (T51-T65) have a 27% increase in odds of a return visit. The remaining risk factors were all blunt force traumas such as injuries to the elbow and forearm (S50-S59); injuries to the wrist, hand, and fingers (S60-S69); and injuries to the hip and thigh (S70-S79). Other trauma-related risk factors (attributable to blunt forces) are shown in Table 3 with corresponding odds ratios and 95% confidence interval.

**Table 3:** Multivariable model

Variables	Levels	OR (95% CI)	*p* value
Injuries/trauma			
Certain early complications of trauma (T79)	–	2.221 (1.998, 2.468)	< 0.001
Burns and corrosions (T20-T32)	–	1.550 (1.454, 1.651)	< 0.001
Other and unspecified effects of external causes (T66-T78)	–	1.547 (1.484, 1.613)	< 0.001
Poisoning by, adverse effect of, and underdosing of drugs, medicaments, and biological substances (T36-T50)	–	1.350 (1.287, 1.416)	< 0.001
Injuries to the head (S00-S09)	–	1.286 (1.258, 1.316)	< 0.001
Toxic effects of substances chiefly nonmedicinal as to source (T51-T65)	–	1.274 (1.191, 1.362)	< 0.001
Injuries to the elbow and forearm (S50-S59)	–	1.203 (1.163, 1.245)	< 0.001
Injuries to the wrist, hand, and fingers (S60-S69)	–	1.180 (1.151, 1.209)	< 0.001
Injuries to the hip and thigh (S70-S79)	–	1.139 (1.098, 1.182)	< 0.001
Injuries involving multiple body regions (T07)	–	1.071 (1.022, 1.123)	0.004
Injury of unspecified body region (T14-T14)	–	1.062 (1.023, 1.104)	0.002
Injuries to the abdomen, lower back, lumbar spine, pelvis, and external genitals (S30-S39)	–	1.038 (1.010, 1.068)	0.008
Injuries to the knee and lower leg (S80-S89)	–	1.035 (1.006, 1.066)	0.018
Injuries to the shoulder and upper arm (S40-S49)	–	0.951 (0.921, 0.981)	0.002
Injuries to the ankle and foot (S90-S99)	–	0.947 (0.919, 0.975)	< 0.001
Injuries to the thorax (S20-S29)	–	0.923 (0.895, 0.951)	< 0.001
Effects of foreign body entering through natural orifice (T15-T19)	–	0.866 (0.800, 0.938)	< 0.001
Demographics, SES, and healthcare utilization			
Length of stay, hours	[0, 1)	Ref	< 0.001
	[1, 12)	1.352 (1.308, 1.398)	
	12 or more	1.742 (1.638, 1.852)	
Payer	Commercial	Ref	< 0.001
	Medicare/Medicaid	1.283 (1.252, 1.314)	
	Other governmental	1.188 (1.131, 1.247)	
	Self-pay	1.225 (1.191, 1.259)	
	Others	1.176 (1.137, 1.216)	
Age (years)	[0, 50)	Ref	
	[50 70)	1.025 (1.003, 1.047)	0.024
	[70 or older)	1.125 (1.092, 1.159)	< 0.001
Race/ethnicity	Caucasian	Ref	
	Hispanic	0.873 (0.815, 0.935)	< 0.001
	African American/Black	0.946 (0.922, 0.971)	< 0.001
	Asian/Pacific Islander	0.916 (0.836, 1.004)	0.062
	Native American	1.036 (0.974, 1.101)	0.262
	Others/Unknown	0.902 (0.870, 0.935)	< 0.001
Sex	Female	Ref	
	Male	1.198 (1.177, 1.219)	< 0.001
	Others/known	0.888 (0.606, 1.300)	0.541
Previous hospitalization (prior 6 months)	0	Ref	< 0.001
	1	1.263 (1.223, 1.305)	
	2	1.369 (1.295, 1.448)	
	3 or more	1.61 (1.512, 1.715)	
Index ED visit is itself a return visit	Yes	1.505 (1.46, 1.551)	< 0.001
Number of previous ED visits (prior 6 months)	0	Ref	< 0.001
	1	1.301 (1.27, 1.333)	
	2	1.655 (1.603, 1.708)	
	3 or more	2.145 (2.078, 2.214)	
Number of previous return visits (prior 6 months)	0	Ref	< 0.001
	1	1.38 (1.326, 1.436)	
	2	2.012 (1.897, 2.135)	
	3 or more	4.014 (3.825, 4.213)	
Diagnoses/comorbidities			
Diseases of the circulatory system (I00-I99)	–	1.032 (1.005, 1.060)	0.020
Disease of the nervous system (G00-G99)	–	1.085 (1.052, 1.118)	< 0.001
Complications of surgical and medical care, not elsewhere classified (T80-T88)	–	1.364 (1.301, 1.43)	< 0.001

We also found that certain injuries/traumas are associated with reduced odds of a return visit. Injuries to the shoulder and upper arm (S40-S49), injuries to the ankle and foot (S90-S99), injuries to the thorax (S20-S29), and effects of foreign body entering through natural orifice (T15-T19) have 5, 5, 8, and 14% decrease in the odds of a return visit. The effect of other trauma variables not captured in Table 2 did not achieve statistical significance.

In addition to these findings on patient demographics, proxies for socioeconomic status, proxies for healthcare utilizations, and certain comorbidities were associated with the risk of a return visit. Older patients have increased odds of a return visit. There is a 20% increase in odds of return visits for male patients compared to female patients. African American/Black patients, as well as patients of Hispanic origins, have 5 and 13% drop in the odds of a return visit compared to Caucasians. Patients with health insurance type other than commercial have increased odds of a return visit. Compared to patients discharged from the ED within the first hour, patients with ED length of stay between 1 and 12 h and those with greater than 12 h length of stay have 35 and 74% increase in odds of a return visit. The number of previous hospitalizations, previous ED visits, and previous return visits to the ED within the last 6 months were all risk factors of a subsequent return visit to the ED. Patients with previous hospitalizations have 26 to 61% increase in odds, those with previous ED visits have 30 to 114% increase in odds, and those with previous return visits have 38 to 301% increase in odds of a subsequent return visit. Furthermore, when a patient experiences a return visit and is discharged home, the odds of a subsequent return visit increases by 50%.

Lastly, patients with comorbidities relating to the circulatory system (I00-I99), the nervous (G00-G99) systems, or arising from complications of surgical and medical care (T80-T88) have 3, 9 and 36% increase in the odds of a return visit respectively.

The AUROC of the mixed-effects model was 0.710 (0.707, 0.712), while the AUROC of the gradient boosting tree ensemble (the machine learning algorithm) was lower at 0.698 CI (0.696, 0.700). In Table 4, we express the performance of the model at specificities between 55 and 95% inclusive. We suggest three risk strata: high risk for patients with predicted probabilities greater than 0.0604 (nearly twice the overall rate of return visits), moderate-risk patients with predicted probabilities between 0.0417 and 0.0604, and low-risk patients with predicted probabilities less than 0.0417 (just slightly higher than the baseline risk). We expect over 50% of all at-risk patients (for return visits to the ED within 72 h) to be captured in the high and moderate risk strata with an overall number needed to evaluate (NNE) of 12.

**Table 4:** Model performance

Specificity (%)	Predicted probability threshold	Sensitivity (%)	PPV	NNE	Risk Strata
95	0.0844	24.7 (24.4, 25.1)	15.9 (15.6, 16.1)	7	High
90	0.0604	36.3 (35.9, 36.7)	12.2 (12.0, 12.3)	9	
85	0.0489	44.5 (44.1, 44.9)	10.2 (10.0, 10.3)	10	Moderate
80	0.0417	50.9 (50.5, 51.3)	8.9 (8.8, 9.0)	12	
75	0.0368	56.2 (55.8, 56.6)	7.9 (7.8, 8.0)	13	Low
70	0.0333	60.9 (60.5, 61.3)	7.2 (7.1, 7.3)	14	
65	0.0307	65.1 (64.7, 65.5)	6.6 (6.6, 6.7)	16	
60	0.0286	69.0 (68.7, 69.4)	6.2 (6.1, 6.2)	17	
55	0.0268	72.7 (72.4, 73.1)	5.8 (5.8, 5.9)	18	

## Limitations

There are, however, some limitations in the data/database used. Proper analyses of the reasons patients return to the ED were not considered given the multi-center nature of the dataset, the absence of clinical notes, and the large sample sizes. We relied on diagnostic codes often riddled with data entry errors and inconsistency of use between providers and institutions. These limitations have a lesser impact as the size of the overall dataset increases. Consequently, we believe that these limitations may have a negligible impact on inference as a result of the very large sample sizes used. Furthermore, variations in clinical care across different EDs in the USA are compensated for using a mixed-effects model with the EDs as random intercepts.

## Discussion/conclusion

ED return visits within 72 h of discharge in adults may be a result of poor quality of care, poor patient education on the use of ED, poor social determinants of health, and complex psychological/psychosocial influences. The underlying causal factors for these return visits have not been formally established, so we rely on statistical associations in order to better identify high-risk patients. In some cases, return visits are unpreventable, such as a patient returning for reasons unrelated to the initial visit, unforeseen deterioration of health unrelated to the quality of care received during the initial visit, and patient misuse of the ED, among others [16–18, 21]. But identification of factors associated with high risk of return visits may help in the identification of high-risk patients for targeted intervention, especially in the presence of scarce and expensive clinical resources for such interventions. In this study, we used mixed-effects regression to explore new variables in search of novel risk factors associated with ED returns for adult patients visiting the ED for trauma (or trauma-related conditions). We assessed the effect of the type of trauma, demographics, and proxies for socioeconomic status, prior ED and hospital utilization variables, diagnoses, and the total number of medications administered during the ED visit.

Our results indicate that both non-blunt and blunt traumas to certain regions of the body are associated with increased odds of a return visit to the ED. The non-blunt traumas include early complications of trauma (such as air/fat embolism, traumatic shock, and traumatic compartment syndrome), burns and corrosions, trauma due to external causes such as hypothermia or asphyxiation, and poisoning resulting by medicaments and biological substances. Factors such as poisoning/adverse effects of medications and nonmedicinal sources and trauma due to external causes (such as hypothermia and asphyxiation) may require a more strategic approach to be impactful.

Blunt traumas to certain body regions of the head, hand, knee, legs, and abdomen/lower back/external genitals were associated with increased odds of a return visit. On the one hand, injuries to the head are often serious and/or alarming due to the potential for death, traumatic brain injury, concussion, and post-concussive syndrome. The gravity or morbidity associated with head injuries may result in higher odds for return visits among patients discharged home. Potential improvement in the quality of care and post-discharge follow-up of patients with head injuries may be achieved with a model such as the mixed-effects model we developed here by careful design of intervention protocols on the discharge education of patients with head injuries. On the other hand, injuries to the hand and regions of the legs may impede mobility and dexterity and are easy to aggravate in the attempt to return to routine daily activities. This is a case where education of patients on the need for rest as well as the risk of a return visit may be helpful. Regardless of the cause, patients with these risk factors may benefit the most from education, social services interventions, and interventions aimed at ameliorating the effects of poor social determinants of health. We note that misdiagnosis captured and left uncorrected in the EMR would be captured in the data used for the study. But the size of the data would guarantee that misdiagnoses are ignorable noise in the study.

The result on prior healthcare utilization variables indicates that patients who may be suffering from complex chronic conditions and/or who have easier access to the healthcare system have higher odds of a return to the ED after the index visit. Patients with chronic conditions are likely to be more educated about the healthcare system (due to frequent utilization), and their return visits are expected to be due to exacerbation of health and unexpected complications due to underlying conditions. These patients are likely to have the highest proportion of unpreventable revisits within 72 h. But this also calls to question the proper management of their chronic conditions and the role of primary care physicians in chronic disease management.

Results on demographics and social determinants indicate that patients from higher socioeconomic families (as captured by health insurance type) have a higher risk of a return visit. While the reason for this association is not clear, we surmise that the use of the ED may be associated with having the means to pay, to be transported, and to spend time away from work or other daily activities. This means that there may be challenges to access of care of patients from lower socioeconomic status. We found a sex difference in the risk of returning to the ED with male patients more likely to have a return visit as well as older patients (compared to their younger peers). The result on difference in sex is expected under the assumption that male patients are more likely to engage in physical (or more physically strenuous) activities that may result in exacerbation of injuries. The result on difference in risk due to age (with older patients more likely to return to the ED) does not lend itself to easy explanations even though we expect that older patients have more complex conditions. We would also expect that older patients are more likely to be admitted to the hospital from the ED, and more care may be taken by providers before discharge home directly from the ED.

The mixed-effects model can be used to rank patients on the predicted probability of returning after discharge. Patients who rank most at-risk for a return visit can be intervened on in any number of the following ways. First, identified risk factors may guide more detailed evaluation of the patient at the initial encounter. Second, additional discharge instructions may be provided based on patient conditions, factors that may result in deterioration of health after discharge, and more detailed post-discharge plans to mitigate unnecessary utilization of the ED. Third, the pre-discharge discussion may facilitate the transition to primary care providers and identify those patients who do not have adequate primary care access. Fourth, proper post-discharge phone calls in cases where appropriate may resolve many issues without unneeded visits while identifying those who need prompt reassessment. These four intervention opportunities are expensive on resources, but targeted interventions based on patients the model predicts to be most at-risk may provide the most impact in the improvement of the overall care of patients.

These risk factors, coupled with the high predictive power of the mixed-effects model (as measured by its AUROC of 0.710), indicate that the model may possess strong clinical utility. The mixed-effects model performed better than the machine learning model, most likely due to the appropriateness of a mixed-effects model in this data/study settings. Most studies on ED return visits have low model performance due to the complexity of reasons for return visits (which may include non-clinical factors not captured in the EMR). Consequently, attention should be paid to the novel variables used in the model in an attempt to improve on existing models. Our findings include novel variables on the type of trauma as well as various patterns of past healthcare utilization. We believe that this model would serve great clinical utility and may help in the identification of proper intervention protocols to reducing unnecessary utilization of the ED. We believe it is of importance in 3 ways: (1) as a simple indication that there are simple risk factors (certain type of traumas) for which the risk of a return visit is high. This informs the ER provider, but the result is a long list that we would not expect providers to memorize among all the important facets for patient care. (2) The findings and corresponding models are meant to be implemented in an electronic and automated system within the EMR. This way, providers do not need to memorize or recall any of the results of the study unless a patient is at high risk of a return visit. Such automated system would include the risk factors contributing to the patient’s high risk. And (3) this work provides incremental addition to literature from which other investigators and researchers can build on.

## Data Availability

The datasets used and/or analyzed during the current study are available from the corresponding author on reasonable request.

## Abbreviations

AUROC: Area under the receiver operator characteristic curve
ICD-10-CM: International Classification of Diseases, Tenth Revision
ED: Emergency department
GVIF: Generalized variance inflation factor
